# Development of the Human–Equine Attachment Scale

**DOI:** 10.1002/evj.70141

**Published:** 2025-12-16

**Authors:** Richard H. Corrigan, Marc Pierard, Emma Davies, David Marlin, Stephanie Evans, Jane M. Williams

**Affiliations:** ^1^ Equine Department Hartpury University Gloucester UK; ^2^ Institute of Health University of Cumbria Carlisle UK; ^3^ School of Veterinary Medicine University of Lancashire Preston UK; ^4^ AnimalWeb Ltd Cambridge UK

**Keywords:** bond, horse, human–horse attachment, psychometrics, scale development, welfare

## Abstract

**Background:**

Human–horse relationships encompass diverse roles, from companion to competition partner. The impact of such bonds informs owner decision‐making regarding horse management and veterinary care, yet standardised instruments to measure these unique bonds are limited.

**Objectives:**

To develop the Human–Equine Attachment Scale (HEAS), a novel instrument to measure the multi‐faceted dimensions of human–horse attachment.

**Study Design:**

Cross‐sectional design using a self‐administered psychometric instrument.

**Methods:**

Initial items were developed through a systematic review of human and animal attachment research, with adaptations made to reflect human–equine relationships. The preliminary scale contained 25 items across five hypothesised factors: Companionship, Wellbeing, Dependence, Status and Growth. Data were collected via an online survey (March–April 2022), recruiting participants through equestrian social media and professional networks using non‐random convenience and snowball sampling.

**Results:**

The final sample comprised 3611 predominantly female (92.9%) respondents. Principal components analysis (PCA) investigated the underlying structure of the scale. The final PCA revealed a six‐factor solution explaining 60% of total variance: Companionship (19%), Personal Wellbeing (9.8%), Dependence (8.9%), Status (8.5%), Growth (7.5%) and Sacrifice (6.3%). The final 22‐item scale demonstrated good internal reliability (Cronbach's *α* = 0.77).

**Main Limitations:**

The self‐report instrument represents UK‐only participants.

**Conclusions:**

The emergence of Sacrifice as a distinct factor highlights unique aspects of horse ownership, particularly regarding financial and personal investment. The HEAS shows promise as a reliable tool for measuring human–horse attachment, with numerous potential applications. It could help bridge the gap in knowledge regarding owner motivation and human–horse relationships, supporting research into how attachment influences welfare, management, and veterinary care decisions. While the scale demonstrates good psychometric properties, further validation across cultural contexts and equestrian populations is recommended. The development of the HEAS represents an important step towards understanding the complex nature of human–horse bonds and their implications for owner decision‐making and practice.

## 1 INTRODUCTION

Companion animals including horses^1^, ^2^ often undertake human‐like roles as ‘family members’^3^, ^4^ across their owners' lifespans.^5^, ^6^ Increasing evidence showcases the strong attachments and bonds owners feel with companion animals^7^, ^8^ and the positive impact on wellbeing including positive child development^9^, ^10^ and emotional regulation in adulthood. Attachment, a human construct applied to human–animal relationships,^11^, ^12^, ^13^, ^14^ demarcates the affectional bond between individuals and a specific attachment figure and includes emotional, cognitive, and behavioural elements.^15^, ^16^ It is influenced by attachment style, personality type, empathy, and species of animal.^17^, ^18^, ^19^, ^20^

Companion animals satisfy the essential features of attachment figures for humans,^18^, ^21^, ^22^ as they contribute to their owners' feeling needed, being accepted, loved, appreciated, and providing comfort, support, and security in times of stress.^23^, ^24^, ^25^, ^26^ Therefore, for many, companion animals represent more than commodities or possessions^27^ and provide social support and positive physical and psychological outcomes.^18^, ^28^, ^29^, ^30^, ^31^ as effectively as significant friends and family members.^18^ Companion animals are also social conduits helping establish human relationships and friendships,^6^, ^32^, ^33^, ^34^ and consequently facilitate positive mental health outcomes.^35^, ^36^

The positive effects of companion animal ownership including horses^37^ or ‘pet effect’^38^ are a burgeoning area of health‐related research.^39^ Attachment is also recognised as a critical component in the veterinary‐client relationship with the level and nature of attachment influencing client decision‐making^40^, ^41^ with positive and negative consequences for pets' quality of life.^18^ Attachment can represent a barrier to adherence to veterinarian advice^42^ for example delaying euthanasia decisions^43^, ^44^ or promote collaborative outcomes that place animals' needs forefront in decision‐making.^45^, ^46^

In the UK, there are ~850,000 horses, 1.82 million people ride regularly, 3.2 million ride occasionally, and a further 6 million households contain former horse riders.^47^ Since domestication, the evolution of the human–horse relationship from primarily owner‐worker to loved companion^48^ influences human interactions and relationships with them.^49^, ^50^

Horses have a unique status as companion animals, as their physiology, functions, and care needs (e.g., size of stabling and turnout) prevent them from being integrated into the internal home life of owners and preclude them from being treated as traditional companion animals.^51^ Horse–human relationships are multifaceted^52^, ^53^ and are influenced by horses' personalities,^54^, ^55^, ^56^ status as friends,^49^, ^51^, ^52^, ^53^, ^54^, ^55^, ^56^, ^57^, ^58^ family,^57^ sporting partners,^52^, ^53^ commodities^53^, ^57^ or service providers.^57^ It is reported that some owners are more bonded to their horse than to other people^59^ and rely on horses to mediate their attachment and emotional needs.^49^, ^60^, ^61^ This suggests that horses are suitable candidates for the formation of human–animal attachment bonds.

Human–animal interactions research evidences the importance of human relationships with companion animals and their significance to human wellbeing.^62^ However, although standardised instruments can provide objective, quantified measurement of attachment from the owners' perspective, many existing instruments contain items that are species exclusive. This limits their scope to capture elements of non‐traditional companion animal connections where there is a utility‐orientated aspect related to working status or financial investment as exist for horses.^63^, ^64^ Uniquely among companion animals, buying, selling, and relocating horses due to financial motivations, human skills and experience, competitive aspirations, lack of time to provide adequate care, or the animal's age and physical capabilities^52^, ^53^, ^57^, ^65^ is commonplace and likely impacts human–horse attachment. This study aimed to develop an instrument capable of capturing the different factors that contribute to owners' attachment to their horses and identify distinctive aspects that are unique to the human–horse relationship.

## 2 MATERIALS AND METHODS

### 2.1 Item development

A systematic review of the literature was conducted to identify instruments and items of potential relevance to human–equine attachment, implementing recognised procedures and utilising a PRISMA framework.^66^ Searches were undertaken across ProQuest, PubMed, Web of Science, PsycINFO, and Google Scholar using combinations of terms relevant to human–animal attachment, bonding, and equine relationships, together with psychometric keywords (e.g., scale, questionnaire, validation). Inclusion criteria required that studies reported the development, validation, or quantitative application of an attachment or bonding scale involving humans and animals. A total of 947 records were initially identified, of which 25 were retained after screening, representing 14 distinct instruments. Across these, five conceptual factors were identified as recurring—Companionship, Wellbeing, Dependence, Status, and Growth.

Six instruments were subsequently selected as the most suitable foundations for the Human–Equine Attachment Scale (HEAS): the Comfort from Companion Animals Scale,^63^ CENSHARE Pet Attachment Scale,^67^ Lexington Attachment to Pets Scale,^68^ Monash Dog–Owner Relationship Scale,^69^ Pet Attachment Questionnaire, and Pet Attachment and Life Impact Scale.^70^ These scales were prioritised for their established reliability, validity, and conceptual relevance to attachment formation in human–equine relationships. This review was undertaken to inform item generation rather than as a standalone publication, and only summary outcomes are therefore reported here.

Items for inclusion in the HEAS were selected from these measures on the basis of their high face validity and alignment with the identified conceptual factors. Using established items facilitated continuity with attachment constructs previously established and utilised in related research; therefore, further consultation with external experts was not considered necessary. This approach is consistent with the methodology frequently employed in companion‐animal attachment scale construction.^63^, ^67^, ^68^

*Factor 1: Companionship*: This factor included items that reflect the friendship, trust, love, and motivation that is derived from the owner–horse relationship. This aligns with the attachment criteria of providing a safe haven and secure‐base.^26^

*Factor 2: Personal wellbeing*: The items in this factor relate to the sense of physical and psychological wellbeing that may result from owner–horse interactions. This integrates items that reflect the perception of the horse as providing perceived enjoyment and comfort, which may result in the desire for proximity maintenance.^71^

*Factor 3: Dependence*: This factor involves the ability of the owner to part with their horse and the potential distress that this may cause. This also aligns with proximity maintenance and separation distress.^14^

*Factor 4: Status*: The items in this factor relate to the extent of challenge that the owner is willing to incur due to the status that they attribute to their horse.^72^

*Factor 5: Growth*: This factor relates to the personal sense of growth that the owner perceives themselves to experience because of their interactions with their horse.^73^, ^74^

#### 2.1.1 Selection of items for inclusion

Following the identification of a pool of potential items from the literature, a structured content validation process was undertaken. Each member of the research panel independently evaluated each potential item for clarity, relevance, and construct fit. Redundant items were then removed, concise wording was prioritised, and the revised set was pilot‐tested for face‐validity.^75^, ^76^, ^77^, ^78^, ^79^ Adaptations were made to the wording of adopted items to reflect the specific focus on human–equine attachment. In extant species‐specific attachment scales, items have been included to reflect the particularities of the human–animal attachment relationship in these species‐specific contexts.^63^, ^69^, ^80^ With this in mind, additional novel items were included to account for the competitive, personal, social, and financial aspects of the human–horse relationship (Table 1).^46^, ^53^, ^57^, ^81^ This resulted in a self‐report instrument comprising 25 positively (*n* = 20) or negatively (*n* = 5) worded items.

**TABLE 1:** Factors, scales and items included in the HEAS.

Factor	Original wording	Original scale	Revised wording
Companionship	I love my pet because he/she is more loyal to me than most of the people in my life	LAPS	I love my horse because he/she is more loyal to me than most of the people in my life.
Companionship	I love my pet because it never judges me	LAPS	My horse makes me feel loved because it never judges me.
Companionship	My pet makes me feel needed	CFCAS	My horse makes me feel needed.
Companionship	My pet makes me feel trusted	CFCAS	My horse makes me feel trusted.
Companionship	I consider my pet to be a friend	LAPS	I consider my horse to be a close friend.
Companionship	Having a pet gives me something to love	CFCAS	Having a horse gives me something to love.
Companionship	My dog gives me a reason to get up in the morning	MDORS	My horse gives me a reason to get up in the morning.
Companionship	You spend time each day grooming your pet	CPAS	I take the time to groom my horse each day.
Wellbeing	My dog helps me get through tough times	MDORS	My horse helps me get through tough times.
Wellbeing	My pet provides me with pleasurable activity.	CFCAS	My horse provides me with pleasurable activity.
Wellbeing	I believe that loving my pet helps me stay healthy	LAPS	Exercise and interaction with my horse helps me stay healthy.
Wellbeing	Owning a pet adds to my happiness	LAPS	Owning a horse increases my happiness.
Wellbeing	I often worry my partner will not want to stay with me	AAS	I worry my horse would do better with another owner.
Dependence		^a^	I will sell/replace my horse when it gets old.
Dependence	I would like to have my dog near me all the time	MDORS	I worry because I cannot spend as much time as I would like with my horse.
Dependence		^a^	I would sell/replace my horse if I could not compete with it.
Dependence	It bothers me that my dog stops me doing things I enjoyed doing before I owned it	MDORS	It annoys me that owning a horse prevents me from doing things I used to enjoy.
Dependence	If necessary, I would be able to give away my pet without any difficulties	PAQ	If necessary, I would be able to give my horse away without any difficulties.
Status		^a^	I would be willing to incur significant financial burden to treat my horse if it was ill or injured.
Status	My dog costs too much money	MDORS	I worry that my horse is costing me too much money.
Status		^a^	Horses deserve as much respect as people do.
Status	I would do almost anything to take care of my pet	LAPS	I would do almost anything to take care of my horse.
Growth		^a^	I am more social because of my horse.
Growth		^a^	I have learned new skills because of my horse.
Growth	I have learned compassion from my pet	PAS	I have learned compassion from my horse.

Abbreviations: CFCAS, Comfort From Companion Animal Scale; CPAS, Censhare Pet Attachment Scale; LAPS, Lexington Attachment to Pets Scale; MDORS, Monash Dog Owner Relationship Scale; PAQ, Pet Attachment Questionnaire; PAS, Pet Attachment and Life Impact Scale.

^a^ New novel items created for inclusion in the HEAS.

A four‐point Likert Scale (1 = Strongly Agree, 2 = Agree, 3 = Disagree, 4 = Strongly Disagree) was adopted as it is an established robust scale of measurement in the social sciences.^82^ By removing the neutral midpoint, participants were encouraged to provide clear directional responses, which enhances the discriminatory power of the data.^83^, ^84^, ^85^ Research has evidenced that implementing positively and negatively worded items results in reduced response set bias, and therefore this was utilised in this study.^86^ Four demographic domains were also surveyed to promote transparency in reporting and position the findings within the context of the study population's characteristics.^87^ They included age, gender, highest level of education, and breed of horse owned.

### 2.2 Sampling and participants

The study adopted a cross‐sectional design that utilised a self‐administered psychometric instrument, the Human‐Equine Attachment Scale, comprising 25 closed Likert‐style questions to measure the extent of human–horse attachment.

The survey was generated using Qualtrics™ and was available for 6 weeks between March and April 2022. The items in the scale were randomly arranged and not ordered or labelled by factor to mitigate response bias.^88^ The ordering of items was identical for all participants. Non‐random convenience and snowball sampling of horse owners and caregivers, and those working in the equine industry was accomplished through posts on Facebook sites, such as Equestrian Southwest and British Eventing, and members of DrDavidMarlin.com. Non‐random, convenience sampling is frequently implemented in research as it allows for rapid collection of data, is inexpensive, and facilitates ease of access to the relevant sample population.^85^ Snowball sampling offers the advantage of increasing participation through inviting professional contacts and participants to share the study with other individuals that satisfy the study's inclusion criteria.^89^ Inclusion criteria limited participation to those who were 18 years of age or older and had either owned or loaned/leased/cared for a horse, or worked within the equine industry with direct human–horse interactions within the last 12 months. The ‘Prevent Multiple Submissions’ feature in Qualtrics was enabled to ensure that each participant could complete the survey only once, thereby avoiding duplicate responses.

### 2.3 Ethical considerations

Hartpury University Ethics Committee granted approval for this study (Ethics number: 2020‐49). Participation was voluntary and respondents were required to indicate their informed consent for their data to be utilised for the purposes of the study.

### 2.4 Data analysis

Data were analysed using SPSS version 26.0 (IBM). A principal components analysis (PCA) was performed to investigate the underlying structure of the 25 Human‐Equine Attachment Scale items. Meyer–Olkin index (KMO) (acceptable minimum value established as 0.60), and Bartlett's test of sphericity (significance set at *p* < 0.05) were utilised to establish the extent of inter‐correlation of items and establish the appropriateness of Factor Analysis.^90^ The 25 item HEAS was then analysed using PCA. Before subjecting data to PCA, they were assessed for accuracy, normality, linearity, multicollinearity, singularity, outliers, and factorability of the correlation matrices. The PCA did not employ factor constraints, and a Varimax rotation was employed that implemented Kaiser normalisation.^91^, ^92^ Following the initial PCA, appropriate adjustments were made to the scale. The process was then reimplemented to establish the ultimate scale.

Eigenvalues were required to be >1.0 and a minimum percentage of variance established at >60%. Loadings of less than 0.4 were suppressed. Cronbach's Alpha score was utilised to establish the internal reliability of the scale. If the scale scored above 0.70, it was considered to have good reliability.^90^, ^93^ The impact on Alpha score following the removal of items was also calculated. Items that failed to satisfy the required threshold were removed, and then analysis was repeated to construct the final scale. After the completion of PCA and reliability testing, the research team analysed factor loadings, cross‐loadings, and item‐total correlations. The statistical findings were considered in combination with theoretical alignment with attachment constructs. Proposed revisions were then discussed collaboratively until agreement was reached. This ensured that the final scale had both conceptual integrity and empirical robustness.

## 3 RESULTS

### 3.1 Participant demographics

There were 4727 respondents, with 1116 being removed due to their data being incomplete. This resulted in a final sample of 3611 respondents (Margin of errors: ±2% at the 99% CI, based on a population of ~5 million people who have regular interactions with horses in the UK (BETA, 2023)). The majority were female (92.9%) followed by male (4.7%) and other/non‐binary (<1%). Most participants were within the 45–54 years age range, followed by 55–64 years (21.4%), with the lowest falling within the 75–84 years range (<1.0%). Approximately a fifth of respondents (22.7%) owned/loaned horse type ‘other’ (which includes all types that are not warmblood, thoroughbred, or Arab), 20.2% owned/loaned warmblood horses, 13.3% owned/loaned Thoroughbreds, with Arabs being the least owned/loaned breed (5.4%). The greatest number (27.6%) of respondents were educated to undergraduate degree level, followed by Further Education (24.0%) with the least number having a doctoral degree (3.7%). For further demographic details, see Tables 2, 3, 4, 5.

**TABLE 2:** Participant gender.

Gender	Frequency	%
Male	168	4.7
Female	3353	92.9
Non‐binary/other	30	0.8
Prefer not to say	60	1.6
Total	3611	100.0

**TABLE 3:** Participant age.

Age	Frequency	%
18–25	257	7.1
25–34	593	16.4
35–44	758	21.0
45–54	889	24.6
55–64	771	21.4
65–74	273	7.6
75–84	19	0.5
Prefer not to say	51	1.4
Total	3611	100.0

**TABLE 4:** Breed owned.

Breed	Frequency	%
Thoroughbred	481	13.3
Warmblood	729	20.2
Thoroughbred Cross	395	10.9
Native	678	18.8
Draught	239	6.6
Arab	194	5.4
Other	821	22.7
Prefer not to say	74	2.0
Total	3611	100.0

**TABLE 5:** Participant education.

Level	Frequency	%
GCSE/school	476	13.2
Further education (a levels/BTEC/equivalent)	868	24.0
Foundation degree	284	7.9
Undergraduate degree	995	27.6
Postgraduate degree	805	22.3
Doctoral degree	132	3.7
Prefer not to say	51	1.4
Total	3611	100.0

### 3.2 Test of adequacy

Bartlett's test of sphericity was statistically significant (*p* < 0.001), which indicated the data were highly likely to be factorisable. There was also superb sampling adequacy (KMO = 0.900). Therefore, PCA was found to be appropriate for the data.

### 3.3 PCA and Cronbach's Alpha

The primary PCA resulted in a 5‐factor solution that explained 51.3% of the total variance.

Cronbach's Alpha test of internal validity (*α* = 0.77) was good. Three items were poorly related to all other items (<0.3) and were therefore removed: ‘I take the time to groom my horse each day’, ‘I worry because I cannot spend as much time as I would like with my horse’ and ‘I worry my horse would do better with another owner’.

Following the removal of these three items, PCA was reimplemented. Visual inspection of the scree plot indicated that it was appropriate to retain all six of the components. Cronbach's Alpha for the total scale was good (*α* = 0.77). Consensus was reached by the research team that the sixth factor should be labelled ‘Sacrifice’, as the items reflected the degree of financial and personal sacrifice that the owner is willing to experience, as a consequence of owning their horse. The results provided a 22‐item solution across 6 factors that explained 60% of the total variance (19.0%: Factor 1—Companionship, 9.8%: Factor 2—Personal Wellbeing, 9.0%: Factor 3—Dependence, 8.5%: Factor 4—Status, 7.5%: Factor 5—Growth and 6.3%: Factor 6—Sacrifice) (Table 6).

**TABLE 6:** HEAS: PCA factor loadings and internal consistency.

		Factor loadings					
Item		Factor 1	Factor 2	Factor 3	Factor 4	Factor 5	Factor 6
2	I love my horse because he/she is more loyal to me than most of the people in my life	0.783					
15	My horse makes me feel loved because it never judges me	0.780					
18	My horse makes me feel needed	0.719					
19	My horse makes me feel trusted	0.682					
16	I consider my horse to be a close friend	0.672					
1	Having a horse gives me something to love	0.668					
17	My horse gives me a reason to get up in the morning	0.610					
22	My horse helps me to get through tough times	0.550	0.408				
4	My horse provides me with pleasurable activity		0.814				
5	Exercise and interaction with my horse helps me stay healthy		0.780				
3	Owning a horse increases my happiness		0.660				
10	*I will sell/replace my horse when it gets old*			0.859			
9	*I would sell/replace my horse if I could not compete with it*			0.848			
12	*If necessary, I would be able to give my horse away without any difficulties*			0.547			
14	I would be willing to incur significant financial burden to treat my horse if it was ill or injured				0.781		
13	Horses deserve as much respect as people do				0.719		
11	I would do almost anything to take care of my horse				0.668		
20	I am more sociable because of my horse					0.739	
6	I have learned new skills because of my horse					0.727	
21	I have learned compassion from my horse					0.603	
8	*I worry that my horse is costing me too much money*						0.869
7	*It annoys me that owning my horse prevents me from doing things I used to enjoy*						0.708
Eigenvalue		6.0	2.1	1.9	1.2	1.0	1.0
Variance (%)		19.0	9.8	9.0	8.500	7.5	6.3
Cumulative variance (%)		19.0	28.8	37.8	46.3	53.7	60.0
Internal consistency (Cronbach's *α*)							0.770

*Note*: Negatively worded items are shown in italics.

## 4 DISCUSSION

This study aimed to develop a new instrument to measure the attachment of horse owners to their horses, the Human‐Equine Attachment Scale (HEAS). Table S1 shows the major statistical analyses regarding validity and reliability for the HEAS and compares them with the existing scales for human–animal attachment that were used as a starting point for the HEAS. The value for Cronbach's Alpha is slightly lower than that reported in some prior studies, and the variance explained is higher than for existing scales, although both exceed acceptable agreement.^90^, ^93^, ^94^, ^95^ This variation could reflect the more diverse non‐traditional human–animal relationship between horses and their keepers, e.g., pet versus professional relationships.

There has been increasing debate considering humans' right to use horses for leisure and recreational purposes as societal views on human–animal relationships evolve and the ethical and moral foundation for common practices is debated.^96^, ^97^, ^98^ As a result, the equestrian sector is being increasingly challenged to be pro‐active regarding maintaining the social licence to operate, which is fundamental for the future of equestrianism and in particular competitive horse sports.^97^, ^98^ While horses as sentient beings retain some element of agency and control over their lives, people ultimately decide the environment they live in and the activities they participate in,^98^ therefore, the welfare of horses is the responsibility of the individuals that manage and interact with them, inferring a duty of care and a vital role in maintaining social licence to operate.^52^, ^97^, ^99^ Research has demonstrated how human attachment can positively and negatively impact decision‐making for other animal species in human care.^100^, ^101^, ^102^ It has been demonstrated that the human–animal bond influences end of life care and veterinary decision‐making in horses^103^; it is logical that the attachment of the human partner is a critical component of this bond and impacts broader management and welfare decisions and therefore underpins social licence to operate. The HEAS can be used as an instrument to assess human‐equine attachment across these contexts, supporting education of veterinary professionals and the dynamics of client‐veterinary interaction.

Differences in the attachment between owners of dogs and cats to their animals have previously been identified.^63^, ^104^, ^105^ Therefore, it is important to measure the attachment of horse owners to their animals separately, using a scale that is adapted to the specificities of how owners keep and use their horses. The HEAS developed within this study provides a tool that can be used for horses and ponies. However, further testing in context is required before it can be used reliably with other *Equidae* such as donkeys, mules, and zebras in zoos that are not identical to horses and ponies in physical or behavioural aspects^105^, ^106^ which could influence how humans experience attachment to them.

Respondent characteristics can influence scores for attachment of people to their pets,^70^, ^107^ as can the personality and behaviour of the individual animal towards humans.^24^, ^108^ Horse–human interaction intrinsically underpins equine management and welfare, leading to the role of horse–human relationships across the diversity of equestrian activities and sports being the topic of much debate.^52^, ^53^ For example, studies evaluating the transition of racehorses from racing to second careers consistently report the relationship with the horse by their owner/keeper as fundamental to a successful relationship.^109^, ^110^ The development of the HEAS can facilitate examination of similar demographic and social effects of humans and horses and has the potential to determine if differences exist within the horse–human bond across different equestrian communities.

In the development of the HEAS, the scope in this study was limited to UK participants who owned a horse or had worked in the equine industry with direct human–horse contact in the last 12 months and were over 18 years old. Even within this population, there was a wide range of self‐reported relationships between humans and horses, which could have an influence on how these humans form attachment bonds with their horses. DeAraugo et al.^58^ suggest that training style could be linked to the attachment of the owner to their horse, with the main finding that the attachment‐avoidance score was higher for participants from the behavioural group, compared to those from the eclectic or conspecific training groups. The HEAS could be used to evaluate how different horse owner characteristics affect how they are attached to their horses and their subsequent influence on owner/keeper decision‐making. Owners who keep a very limited number of horses for many years could be contrasted with staff working at a sales yard where horses only reside for a limited time and with a high turnover of horses, for example. It will also be relevant to study the effect of cultural differences on how owners attach to their horses, for example by comparing the responses from different countries or subpopulations with different cultural backgrounds within the same country.^111^ Cautious interpretation of results would be required if the HEAS is to be used for owners whose first language is not English because they may interpret the questions slightly differently or there may be different nuances in some translated versions of the questionnaire.^112^ Being aware of potential cross‐cultural differences does not preclude cross‐cultural similarities, as demonstrated by Haddy et al.^113^ who showed that owner attitudes and their belief in the sentience of their working equids was linked to the welfare of their horses, mules, and donkeys across six countries on four continents.

The current phrasing of the questions within the HEAS is focused on owners of horses or at least people who have the power to decide what happens to the horse: the horses' keeper. This could make some of the questions in the questionnaire in its current form hard to answer for people who have no say in the management of the horse, for example, people who ride horses in a riding school, grooms, or participants in equine assisted activities. To fully evaluate their attachment to particular horses, it could be useful to look at an alternative phrasing for those questions that specifically ask about management decisions. Alternative versions of the questionnaire have been proposed in Table S2, one to be used with people who work closely with horses without owning them (e.g., grooms, volunteers), and one for riders who do not own the horses they ride, and one for people who participate in equine assisted activities. These alternative versions have not been tested to date but could help to expand the scope of the HEAS and help support investigation into the duration and frequency of horse interactions required to initiate a bond and how these bonds develop over time. For example, Törmälehto and Korkiamäki^22^ showed that adolescents expressed feelings consistent with an attachment to the horses after only one visit based on extensive discussions with the participants. This approach requires much more time to analyse compared to a questionnaire like the HEAS; future work to compare validated HEAS outcomes to in‐depth qualitative methodologies could support this to allow much easier collection of larger amounts of data.

The attachment style humans have towards other humans could affect human–animal relationships. The relationship horse owners have with their veterinarian can contribute to trust promoting collaborative decision‐making, increase positive engagement with the provision of prophylactic care, and informed management and end‐of‐life decision‐making for older horses; factors which impact the quality of life of the animals affected.^103^ Adolescents at risk for mental health and behavioural difficulties influenced the behaviour and physiology of horses being groomed,^114^ which in turn could influence how these humans perceive the horses. Similar challenges are reported within dog ownership; Gobbo and Zupan^115^ showed that the attachment style of owners to their dogs had an effect on the aggression level of their dogs. While Lefebvre et al.^116^ found a link between housing and handlers practising sports with their military working dogs and the aggression and welfare of those dogs. Working as an equine veterinarian carries a high risk of occupational injury^117^ with a recent survey^118^ reporting 95% of practitioners surveyed regularly worked with ‘difficult’ horses. It would be useful to test if the effects of human attachment on canine behaviour observed in dogs also exist for horses and, if so, how this can be used to induce human behaviour change in the equine industry to increase safety for veterinarians and other professionals, improve preventative care and management practices by owners to optimise equine welfare.

The decision to recruit participants through DrDavidMarlin.com gave rise to a potential limitation, as the site has been developed and maintained by an evidence‐based equine physiologist and may therefore be more representative of a sub‐population that has a stronger scientific or evidence‐based orientation. Therefore, this recruitment channel may have resulted in some bias by over‐representing participants with research‐informed views rather than those fully reflective of the wider equestrian population. A further methodological limitation of this study is that intra‐rater repeatability was not assessed through re‐administration of the measure at a later point in time. Evaluating test–retest reliability is valuable for confirming response stability; therefore, future research should assess temporal consistency to strengthen the overall validation of the scale.

## 5 CONCLUSION

With horses in industrialised countries moving ever more from working animals to sports and companion animals, understanding the emotional bond between humans and their horses is increasingly important. This study showed that a specific tool to reliably measure attachment from humans towards their horses is feasible. The Human Equine Attachment Scale explained 60% of the variance and captured six factors of the human–horse bond. The HEAS can be used to increase understanding of horse–human relationships and the influence of these on management and veterinary decision‐making. The results could be applied to underpin intervention design for effective human behaviour change aimed at optimising prophylactic veterinary care and horse welfare. There is also scope to adapt the wording of items in the scale to facilitate its application across diverse groups, such as those participating in equine‐assisted therapy, those working in the equine industry, and non‐owner riders.

## FUNDING INFORMATION

No funding was received for this research.

## CONFLICT OF INTEREST STATEMENT

The authors declare no conflicts of interest.

## AUTHOR CONTRIBUTIONS

**Richard H. Corrigan:** Conceptualization; investigation; writing – original draft; methodology; writing – review and editing; software; formal analysis; data curation; project administration. **Marc Pierard:** Conceptualization; investigation; writing – original draft; writing – review and editing. **Emma Davies:** Writing – review and editing. **David Marlin:** Writing – review and editing; data curation. **Stephanie Evans:** Conceptualization; investigation. **Jane M. Williams:** Conceptualization; investigation; writing – original draft; writing – review and editing; project administration.

## DATA INTEGRITY STATEMENT

Richard H. Corrigan had full access to all the data in the study and takes responsibility for the integrity of the data and the accuracy of the data analysis.

## ETHICAL ANIMAL RESEARCH

Hartpury University Ethics Committee granted approval for this study (Ethics number: 2020‐49).

## INFORMED CONSENT

Completion of the psychometric instrument was taken as consent for participation.

## Supporting information


**Table S1.** Comparison of HEAS to other attachment scales used to develop it.

**Table S2.** Alternative wording for the HEAS for different contexts.

## Data Availability

The data that support the findings of this study are openly available on the Open Science Framework repository at https://doi.org/10.17605/OSF.IO/7KA49, reference number 7ka49.
