# Supplementary material for: Development of the Human–Equine Attachment Scale
Source: Equine Vet J. 2025 Dec 16;58(5):1308–18. doi: 10.1002/evj.70141 (PMC13447911; doi:10.1002/evj.70141)
Supplement: Supplementary file 1 [file EVJ-58-1308-s001.pdf]

Table S2: Alternative wording for the HEAS for different contexts.

Item No.	Human Equine Attachment Scale (HEAS)	Scale Domain	Wording for Equine Assisted Therapy Relationships	Equine Worker Relationships	Non-Owner Rider Relationships with Favourite Horse
1	Having a horse gives me something to love.	Companionship	Being able to spend time with my therapy horse gives me something to love.	Working with horses gives me something to love.	Having a horse gives me something to love.
2	I love my horse because he/she is more loyal to me than most of the people in my life.	Companionship	I love my therapy horse because he/she is more loyal to me than most of the people in my life.	I love the horses I work with because they are more loyal to me than most of the people in my life.	I love my horse because he/she is more loyal to me than most of the people in my life.
3	Owning a horse increases my happiness.	Wellbeing	Participating in therapy with my therapy horse increases my happiness.	The horses I work with increase my happiness.	Having a horse increases my happiness.
4	My horse provides me with pleasurable activity.	Wellbeing	My therapy horse provides me with pleasurable activity.	The horses I work with provide me with pleasurable activity.	My horse provides me with pleasurable activity.
5	Exercise and interaction with my horse helps me stay healthy.	Wellbeing	Exercise and interaction with my therapy horse helps me stay healthy.	Exercise and interaction with the horses I work with helps me stay healthy.	Exercise and interaction with my horse helps me stay healthy.
6	I would do almost anything to take care of my horse.	Status	I would do almost anything to take care of/spend time with my therapy horse.	I would do almost anything to take care of the horses I work with.	I would do almost anything to take care of my horse.
7	I have learned compassion from my horse.	Growth	I have learned compassion from my therapy horse.	I have learned compassion from the horses I work with.	I have learned compassion from my horse.
8	I have learned new skills because of my horse.	Growth	I have learned new skills because of my therapy horse.	I have learned new skills because of the horses I work with.	I have learned new skills because of my horse.

9	I would sell/replace my horse if I could not ride/compete/drive with it.	Dependence	I would be upset if I could no longer have therapy with my usual therapy horse.	I think it is ok to sell horses when they are no longer capable of being ridden/driven/used for competition.	I would replace my horse if I could not ride/compete/drive with it.
10	I will sell/replace my horse when it gets old.	Dependence	I would no longer want to have therapy with my horse if he/she got old.	It does not bother me if the horses I work with are sold because they have gotten old.	I will replace my horse when it gets old.
11	I worry that my horse is costing me too much money.	Sacrifice	I worry that attending therapy with my therapy horse involves too much effort.	I worry that working with horses is having a negative impact on my financial situation.	I worry that my horse is costing me too much money.
12	It annoys me that owning a horse prevents me from doing things I used to enjoy.	Sacrifice	It annoys me that spending time in therapy with my therapy horse prevents me from spending time doing things I would enjoy more.	It annoys me that working with horses prevents me from doing other work I would enjoy more.	It annoys me that having a horse prevents me from doing things I used to enjoy.
13	I would be willing to incur significant financial burden to treat my horse if it was ill or injured.	Status	I would be willing to expend significant effort to ensure my therapy horse was treated if it was ill or injured.	I would be willing to expend significant effort to ensure my favourite horse was treated if it was ill or injured.	I would be willing to incur significant financial burden to treat my horse if it was ill or injured.
14	If necessary, I would be able to give my horse away without any difficulties.	Dependence	If necessary, I would be able to be able to leave my therapy horse without any difficulties.	If necessary, I would be able to be able to leave the horses I work with without any difficulties.	If necessary, I would be able to give my horse away without any difficulties.
15	My horse makes me feel loved because it never judges me.	Companionship	My therapy horse makes me feel loved because they never judge me.	The horses I work with make me feel loved because they never judge me.	My horse makes me feel loved because it never judges me.
16	I consider my horse to be a close friend.	Companionship	I consider my therapy horse to be a close friend.	I consider the horses I work with to be close friends.	I consider my horse to be a close friend.

17	My horse gives me a reason to get up in the morning.	Companionship	Visting my therapy horse gives me a reason to get up in the morning.	The horses I work with give me a reason to get up in the morning.	My horse gives me a reason to get up in the morning.
18	My horse makes me feel needed.	Companionship	My therapy horse makes me feel needed.	The horses I work with make me feel needed.	My horse makes me feel needed.
19	My horse makes me feel trusted.	Companionship	My therapy horse makes me feel trusted.	The horses I work with make me feel trusted.	My horse makes me feel trusted.
20	Horses deserve as much respect as people do.	Status	Horses deserve as much respect as people do.	Horses deserve as much respect as people do.	Horses deserve as much respect as people do.
21	I am more social because of my horse.	Growth	I am more social because of my therapy horse.	I am more social because of the horses I work with.	I am more social because of my horse.
22	My horse helps me get through tough times.	Companionship	My therapy horse helps me get through tough times.	The horses I work with help me get through tough times.	My horse helps me get through tough times.
