# Supplementary material for: Development of the Human–Equine Attachment Scale
Source: Equine Vet J. 2025 Dec 16;58(5):1308–18. doi: 10.1002/evj.70141 (PMC13447911; doi:10.1002/evj.70141)
Supplement: Supplementary file 2 [file EVJ-58-1308-s002.pdf]

Equine Veterinary Journal BEVA

Table S1: Comparison of HEAS to other attachment scales used to develop it.

Scale			Abbreviation	Reference
Pet Attachment Questionnaire	PAQ	72	Cronbach's Alpha	PCA
Adult Attachment Scale	AAS	Collins and Read, 1996	0.86 and 0.87	2 factors; 26 items; 41% variance explained
Lexington Attachment to Pets Scale	LAPS	Johnson et al., 1992	0.69, 0.75 and 0.72 for the prototypical descriptions	3 prototypical descriptions, 18 items
Comfort From Companion Animal Scale	CFCAS	66	0.928	3 factors; 23 items; 53.5% variance explained
Censhare Pet Attachment Scale	CPAS	Holcomb et al., 1985	0.85	No PCA reported, 13 items used
Monash Dog Owner Relationship Scale	MDORS	71	0.9	
Pet Attachment and Life Impact Scale	PALS	73	0.84 for each factor of part II; 0.67 for part I	Part I 1 factor with 9 items and 20% variance explained; Part II 2 factors with 19 items and 30% variance explained
Human-Equine Attachment Scale	HEAS		Not reported	4 factors with 35 items, 56.77% variance explained
